# Amygdalo‐nigral circuit mediates stress‐induced vulnerability to the parkinsonian toxin MPTP

**DOI:** 10.1111/cns.14151

**Published:** 2023-03-13

**Authors:** Hongwei Cai, Pei Zhang, Tongxia Li, Ming Li, Lijun Zhang, Chi Cui, Jie Lei, Jian Yang, Kun Ren, Jie Ming, Bo Tian

**Affiliations:** ^1^ Department of Neurobiology, School of Basic Medicine, Tongji Medical College Huazhong University of Science and Technology Wuhan Hubei China; ^2^ Clinical College of Traditional Chinese Medicine Hubei University of Chinese Medicine Wuhan Hubei China; ^3^ Institute for Brain Research Huazhong University of Science and Technology Wuhan Hubei China; ^4^ Key Laboratory of Neurological Diseases, Ministry of Education Wuhan Hubei China; ^5^ Department of Breast and Thyroid Surgery, Union Hospital, Tongji Medical College Huazhong University of Science and Technology Wuhan Hubei China

**Keywords:** amygdalo‐nigral circuit, dopaminergic neuronal death, parkinsonian toxin MPTP, social defeat stress

## Abstract

**Aims:**

The aim was to investigate the effect of mood disorders on parkinsonian toxin 1‐methyl‐4‐phenyl‐1,2,3,6‐tetrahydropyridine (MPTP)‐induced motor disability, substantia nigra pars compacta (SNc) dopaminergic (DA) neurons loss. Also, the neural circuit mechanism was elucidated.

**Methods:**

The depression‐like (physical stress, PS) and anxiety‐like (emotional stress, ES) mouse models were established by the three‐chamber social defeat stress (SDS). The features of Parkinson's disease were reproduced by MPTP injection. Viral‐based whole‐brain mapping was utilized to resolve the stress‐induced global changes in direct inputs onto SNc DA neurons. Calcium imaging and chemogenetic techniques were applied to verify the function of the related neural pathway.

**Results:**

We found that PS mice, but not ES mice, showed worse movement performance and more SNc DA neuronal loss than control mice after MPTP administration. The projection from the central amygdala (CeA) to the SNc^DA^ was significantly increased in PS mice. The activity of SNc‐projected CeA neurons was enhanced in PS mice. Activating or inhibiting the CeA‐SNc^DA^ pathway could mimic or block PS‐induced vulnerability to MPTP.

**Conclusions:**

These results indicated that projections from CeA to SNc DA neurons contribute to SDS‐induced vulnerability to MPTP in mice.

## Graphical abstract

Physical stress mice, but not emotional stress mice, are susceptible to MPTP. The projection from CeA to the SNc^DA^ was significantly increased in Physical stress mic. The activity of SNc‐projected CeA neurons was enhanced in Physical stress mice. Activating or inhibiting the CeA‐SNc^DA^ pathway could mimic or block PS‐induced vulnerability to MPTP.

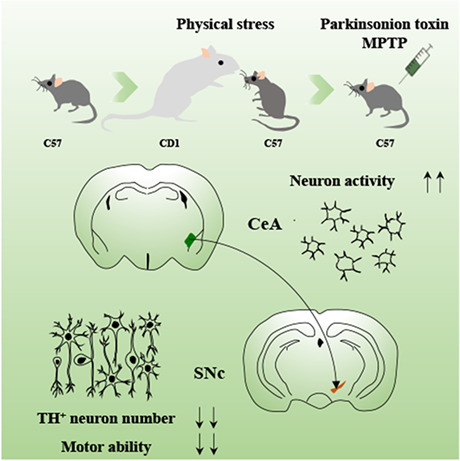

## 1 INTRODUCTION

Parkinson's disease (PD) is a degenerative disease of the central nervous system, and the most obvious symptoms are movement disorders such as shaking, rigidity, slowness of movement, and difficulty walking and gait. Later, it can cause neuropsychiatric disturbances that range from mild to severe, including disorders of speech, cognition, thought, and mood.^1^ As PD advances, these nonmotor symptoms can be predominant.^2^ In the experiment, MPTP is one of the most accepted neurotoxins widely utilized to induce the PD mouse model nowadays.^3^ Both acute and chronic stress play an important role in PD.^4^ There is a great deal of clinical evidence that stress can increase the symptoms of PD.^5^ It has been reported that PD patients may find that their tremor is worsened when they become anxious or angry.^6^ Stress‐induced toxicity has been shown in many diseases, including schizophrenia, stroke, Alzheimer's disease, anxiety, and depression.^7^ More recently, several authors have speculated about the role of emotional stress (ES) in PD.^8^, ^9^, ^10^ A review revealed that enhanced psychological stress during a pandemic can disrupt several motor functions.^11^ Besides, a survey of 5000 patients revealed that PD patients experience greater levels of stress than controls and that stress worsens both motor and nonmotor symptoms.^12^ On the basis of these theories, we hypothesize that stress‐induced mood disorders contribute to the progression of PD.

Stress‐induced depressive disorders and anxiety disorders are common neuropsychiatric diseases worldwide that limit psychosocial functioning and diminish the quality of life. Mood disorders and PD share some common pathophysiological features. In our previously published study,^13^ we observed that the mice in the physical stress (PS) group showed depression‐like behavior, and others in the ES group manifested anxiety‐like phenotype after treating with the three‐chamber social defeat stress (SDS) procedures. Thus, this model provides the opportunity for studying simultaneously the impact of stress‐induced depressive disorders and anxiety disorders on PD. The basal ganglia, a group of brain structures innervated by the dopaminergic system, are the most seriously affected brain areas in PD.^14^ The main pathological characteristic of PD is selective loss of dopaminergic (DA) neurons in the substantia nigra pars compacta (SNc),^15^, ^16^ affecting up to 30% of the DA cell bodies and approximately 50% of the DA fibers by the time of death.^17^ A hypothesis of the cause of depression was a deficiency in monoamine neurotransmitters, which consist of serotonin, norepinephrine, and dopamine. It has been implicated in both major depressive disorder^18^ and PD.^19^ Studies have also revealed early‐phase dysfunction of SNc DA neurons and impairment of cortico‐striatal long‐term depression in PD transgenic mice.^20^ Besides SNc, it was reported that the spread of pathologic α‐synclein was also observed in amygdala.^21^ Amygdala is a region of the brain primarily associated with emotional processes. The previous study has found amygdala changes in emotionality in people suffering from PD.^22^ It is generally considered that the central nucleus of amygdala (CeA) functions as a major output of the amygdala by converging inputs from the basolateral nucleus and other amygdalar subregions during the information processing after stress.^23^ Thus, we wonder if amygdalar subregion, such as CeA, was also involved in the effect of stress‐induced mood disorders on PD. These findings show the interdependence between mood disorders and advancing PD. However, it remains unclear how stress‐induced mood disorders affect PD.

The aim of this study was to characterize the role of stress‐induced mood disorders in the progression of PD and the neural mechanism. First, we utilized the three‐chamber SDS procedures to establish depression‐like (PS group) and anxiety‐like (ES group) mouse models. Then, a series of behavioral tests were operated to measure motor disability caused by MPTP. Meanwhile, immunofluorescence staining was used to detect DA neurons death in SNc. Next, whole‐brain retrograde virus tracing and the GCaMP6s technique were applied to probe potential neural circuits underlying stress‐induced vulnerability to the parkinsonian toxin MPTP at the anatomical and functional levels. Finally, we adopted chemogenetic manipulation in vivo to further verified the adequacy and necessity of this neural pathway.

## 2 METHODS

### 2.1 Animals

Adult male C57BL/6J mice (8 weeks) and CD1 mice (4–6 months) were used in this study. C57BL/6J mice were group‐housed and CD1 mice were single‐housed before experiments under a constant temperature of 22 ± 1°C in a 12‐h light/dark cycle with free access to fresh food and clean water. Experimental procedures were approved by institutional guidelines and the Animal Care and Use Committee (Laboratory animal center of Huazhong University of Science and Technology, Wuhan, China).

### 2.2 Three‐chamber social defeat stress mouse model

SDS protocol was carried out according to Golden et al.^24^ and Guangjian Qi et al.^13^ Prior to experiments, CD1 were selected as aggressors based on two criteria: CD1 attack at least two consecutive sessions in 180 s during 3 consecutive days; the latency to initiate aggression must be less than 60 s. Then, SDS experiment was administrated as follows: (i) in the three‐chamber cage, CD1 was placed in the middle chamber, between the PS mouse and PS mouse, separated with a perforated transparent partition and (ii) exposing intruder ES mouse to CD1 aggressor in the middle chamber. After 5 min of social defeat, transfer the ES mouse back to the left chamber for the remainder of 24 h. The ES mouse was staying in the right chamber and observed the whole procedure without receiving direct physical contact; and (iii) repeating SDS for successive 10 days. For each subsequent daily 5 min defeat, CD1 aggressors were not removed from their chamber, but PS and ES mice were alternated daily to prevent habituation to the same CD1. Without any physical or emotional stimuli, the control mouse (CON) was housed in the left or right chamber with CD1 in the middle chamber for 10 days.

### 2.3 MPTP mouse model

The MPTP model was carried out on the basis of the previous study.^25^ According to the paradigm, mice received MPTP (25 mg/kg, i.p.) or equal amounts of 0.9% saline (NS, i.p.) injection for 7 consecutive days. Behavioral performances were assessed in the locomotion test, rotarod test, pole test, and edge test. Neuronal death was detected by immunofluorescence staining 7 days after MPTP injection.

### 2.4 Viral‐based whole‐brain mapping

Totally 150 nL of AAV9‐TH‐Cre, AAV9‐DIO‐TVA‐EYFP, and AAV9‐DIO‐G were mixed in 3:1:2 and injected into left SNc. Once the helper virus reach the expression peak, the retrograde virus RV‐EnVA‐ΔG‐dsRed were injected in the same site with a mass of 150 nL. After 10 days of expression, mice were sacrificed to remove the brains. All brains were fixed in 4% paraformaldehyde and dehydrated with 20% and 30% sucrose. Then, brains were sliced in 30 μm at nine representative coronal levels from bregma: +2.0, +1.5, +0.5, −0.5, −1.2, −2.0, −3.0, −4.0, −5.6, and −6.6 mm. Next, all the slices were imaged via an automatic scanning fluorescence microscope (Olympus, SV120). Finally, neurons marked by fluorescent proteins in corresponding upstream brain regions were calculated by Image J (Fiji). All brain regions we analyzed included the Primary motor area (MOp), Nucleus accumbens (ACB), Caudoputamen (CP), Substantia innominate (SI), External segment of Globus pallidus (GPe), Central amygdala nucleus (CeA), Paraventricular hypothalamic nucleus (PVH), Zona incerta (ZI), Reticular part of Substantia nigra (SNr), Periaqueductal gray (PAG), Motor related Superior colliculus (SCm), Inferior colliculus (IC), Parabrachial nucleus (PB), and Interposed nucleus (IP), which was aligned by the mouse brain map of Allen Brain Atlas.

### 2.5 In vivo fiber photometry

In the in vivo fiber photometry experiment, we first injected 300 nL mixed helper virus of AAV9‐TH‐Cre, AAV9‐DIO‐G, and AAV9‐DIO‐TVA‐His‐EYFP into bilateral SNc (150 nL/unilateral). After SDS administration, an equal amount of RV‐EnVA‐ΔG‐GCaMP6s‐dsRed was injected into the same site. Meanwhile, optical fibers were planted 0.5 mm above the coordinate of bilateral CeA (from bregma: AP ±2.85 mm, ML −1.4 mm, DV ‐4.23 mm). After 10 days, the retrograde virus was fully expressed. When mice undergo the SI test, the calcium activity of neurons in CeA that project to SNc TH^+^ cells was synchronously recorded through the fiber‐photometry system (Inper Technology Co., Ltd). The raw Ca^2+^ fluorescence data was coupled with the track of mice in the SI test and then normalized by z‐score analysis. Finally, the images and results were expressed through MATLAB.

### 2.6 In vivo chemogenetic manipulation

For the hM4Di‐inhibited manipulation experiment, mice were injected 150 nL AAV1‐TH‐Cre into bilateral CeA and 300 nL AAV‐DIO‐hM4Di‐mCherry into bilateral SNc, or 300 nL AAV‐DIO‐mCherry into bilateral SNc as a comparison. During the 10‐day SDS molding treatment, mice received a daily injection of Clozapine‐N‐Oxide (CNO, 3 mg/kg, i.p.) or an equal amount of 0.9% saline (NS, i.p.). For the hM3Dq‐activated manipulation experiment, mice were injected 150 nL AAV1‐TH‐Cre into bilateral CeA and 300 nL AAV‐DIO‐hM3Dq‐mCherry into bilateral SNc, or 300 nL AAV‐DIO‐mCherry into bilateral SNc as a comparison. It took 21 days for viruses to get the peak of expression. Then, mice did not accept SDS molding treatment but received a daily injection of CNO (3 mg/kg, i.p.) and NS (i.p.) 30 min before MPTP operation for 7 consecutive days. The statistical values are reported in Supplementary Table S1. Meanwhile, the detailed methods are provided in the Supplementary Appendix S1.

## 3 RESULTS

### 3.1 PS aggravates MPTP‐induced movement disorders and SNc DA neuronal loss

**FIGURE 1:**
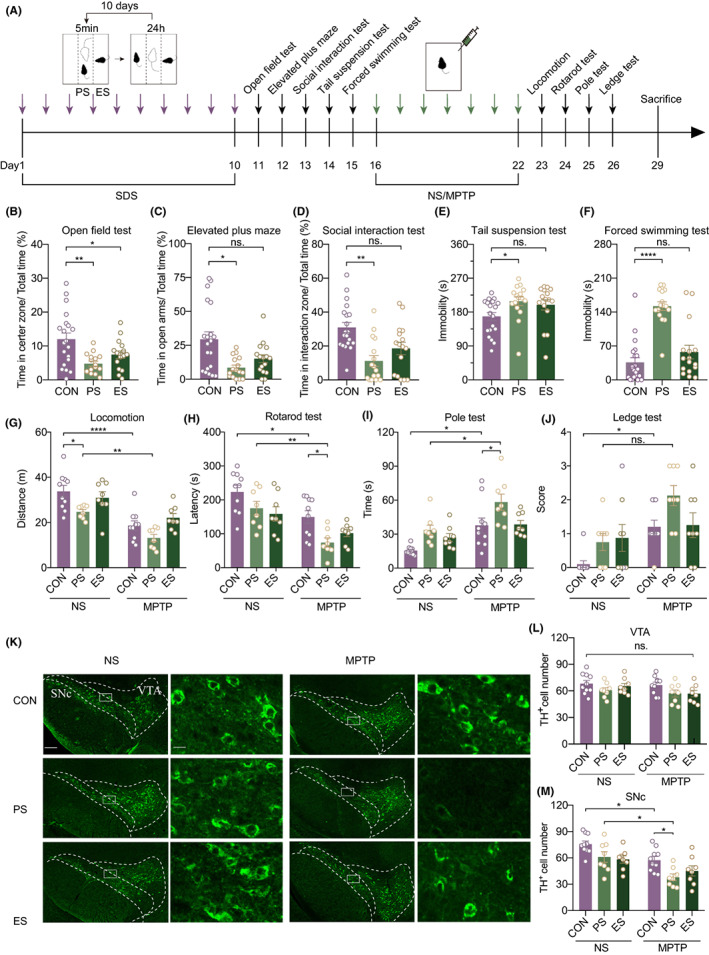
Physical stress aggravates MPTP‐induced movement disorders and TH^+^ neurons death in SNc. (A) Experiment scheme for MPTP model with mood disorders. The purple arrows represented daily SDS modeling and the green arrows represented daily saline or MPTP injection. (B–F) Behavioral assessments of three‐chamber SDS in individual groups. (B) Percentage of time in the center zone of an open field test. (C) Percentage of time in open arms of the elevated plus maze. (D) Percentage of time in interaction zone of social interaction test. (E) Immobility time in tail suspension test. (F) Immobility time in a forced swimming test. (G–N) Behavioral evaluation and neural death induced by MPTP in individual groups. (G) Total distance traveled in locomotion test. (H) Latency to fall from rotarod. (I) Time for mice to get from the top down to the bottom in the pole test. (J) Score of mice in ledge test. (K) Representative images of TH^+^ neurons in VTA and SNc at low and high magnification. Scale bar = 150/15 μm. (L) TH^+^ cell calculation in VTA of individual groups. (M) TH^+^ cell calculation in SNc of individual groups.

To explore the potential effects of mood disorders on MPTP‐induced damage, we employed a 10‐day consecutive three‐chamber SDS mouse model (Figure 1A). The PS mouse in the left chamber was introduced to the middle chamber to physically contact the aggressive CD1 mouse for 5 min and go through attack. Without direct physical stimuli, the ES mouse in the right chamber was subjected to scene‐dependent emotional stimulation by observing the PS mouse receiving social defeat. After the modeling operation, a series of behavioral tests were performed to assess SDS modeling. The open field test and elevated plus maze test were conducted to assess anxiety‐like behavior. The time PS mice and ES mice spent in the center zone (CON vs. PS, *p* = 0.0010; CON vs. ES, *p* = 0.0432) were both decreased relative to CON mice in the open field test (Figure 1B). PS mice also spent less time in the open arm (CON vs. PS, *p* = 0.004; CON vs. ES, *p* = 0.406) than CON mice in the elevated plus maze test (Figure 1C). A social interaction test was used to measure SDS‐induced social avoidance. Resistant mice (social interaction ratio >1) were excluded from all experiments. The results showed that PS mice and ES mice exhibited a smaller interaction zone (CON vs. PS, *p* = 0.001; CON vs. ES, *p* = 0.014) with the appearance of CD1 than CON mice (Figure 1D). The tail suspension test and forced swimming test were performed to evaluate depressive behavior of despair. The PS mice displayed more immobility time (CON vs. PS, *p* = 0.019; CON vs. ES, *p* = 0.060) in both tests, but ES mice showed immobility (CON vs. PS, *p* =0.000076; CON vs. ES, *p* = 1.000) levels similar to those of CON mice (Figure 1 E,F). Together, these results demonstrated that three‐chamber SDS induced reliable depressive‐like behaviors and typical anxiety‐like behaviors.

Next, we wanted to determine whether SDS influenced the motor disability induced by MPTP. Following the forced swimming test, mice received 7‐day consecutive MPTP intraperitoneal injections at a dose of 25 mg/kg daily, while the control mice received equal 0.9% saline (NS) intraperitoneal injections. Twenty‐four hours after the last dose, a series of behavioral tests were conducted to estimate the movement ability of all mice. Then, the mice were sacrificed 7 days after the last dose to detect the numbers of tyrosine hydroxylase (TH)‐positive neurons in the SNc (Figure 1A). In the locomotion test, PS mice traveled less distance than control mice in the NS group, which implied the efficacy of SDS. In addition, the distance (CON + NS vs. CON+MPTP, *p* = 0.0441; CON + NS vs. PS + MPTP, *p* < 0.0001; PS + NS vs. PS + MPTP, *p* = 0.0080) was decreased in both control mice and PS mice that accepted MPTP treatment compared with those that accepted NS injection (Figure 1G). The rotarod test is widely used to evaluate MPTP models. We found that the latencies to fall from the rotarod were obviously shortened in CON and PS mice with MPTP treatment relative to NS treatment. More importantly, PS mice exhibited less latency (CON + NS vs. CON + MPTP, *p* = 0.0330; PS + NS vs. PS + MPTP, *p* = 0.0053; CON + MPTP vs. PS + MPTP, *p* = 0.0483) than CON mice in the MPTP group (Figure 1H). Consistent with the rotarod test, the performance of CON and ES mice injected with MPTP was worse in the pole test (CON + NS vs. CON + MPTP, *p* = 0.0136; PS+ NS vs. PS + MPTP, *p* = 0.0140; CON + MPTP vs. PS + MPTP, *p* = 0.0434), especially PS mice (Figure 1I). In the ledge test (CON + NS vs. CON + MPTP, *p* = 0.047; PS + NS vs. PS + MPTP, *p* = 0.152; CON + MPTP vs. PS + MPTP, *p* = 1.000), CON but not PS and ES mice injected with MPTP got lower score (Figure 1J). Tyrosine hydroxylase was accepted as a marker of DA neurons. Through TH^+^ neuron immunolabeling and analyzing, we found the number of TH^+^ neurons in the VTA (CON + NS vs. CON + MPTP, *p* = 0.9992; PS + NS vs. PS + MPTP, *p* = 0.9795; CON + MPTP vs. PS + MPTP, *p* = 0.3794) was not different among the six groups (Figure 1K,L). However, in the SNc, the death of TH^+^ neurons (CON + NS vs. CON + MPTP, *p* = 0.0419; PS + NS vs. PS + MPTP, *p* = 0.0148; CON + MPTP vs. PS + MPTP, *p* = 0.0401) occurred in all MPTP‐operated animals except ES mice. PS mice showed even lower levels than CON mice (Figure 1K,M). Surprisingly, ES mice showed no significant changes between the NS group and MPTP group through the above behavioral tests and cell calculations. This result indicated that animals with anxiety were not susceptible to MPTP. Taken together, these results suggested that PS could aggravate movement disorders and TH^+^ neuron death caused by MPTP.

### 3.2 Mapping global changes in inputs to SNc DA neurons in depressed mice

**FIGURE 2:**
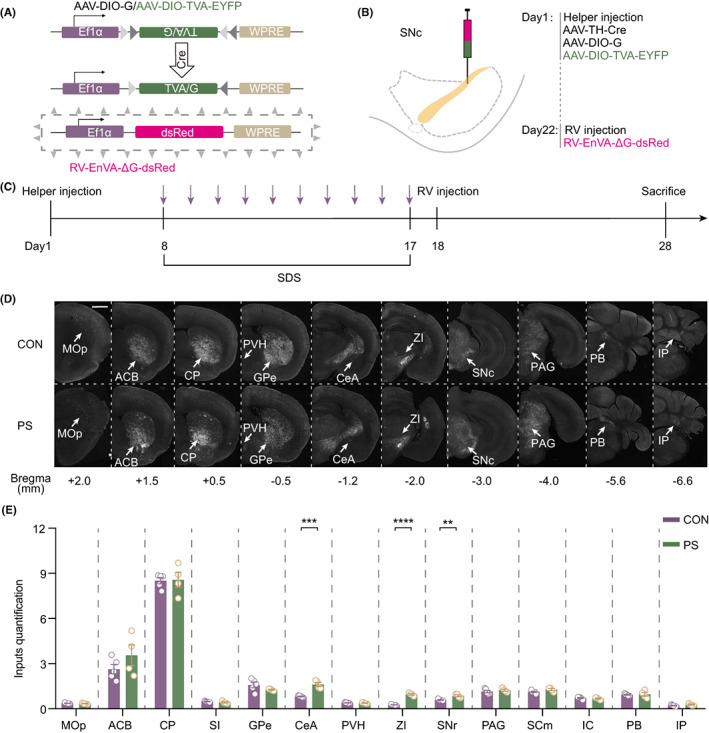
Mapping global changes of inputs to SNc DA neurons in PS mice. (A) Schematic representation of virus vector construction. (B) The strategy of viral injection. (C) Schematic representation of experiment procedure. The purple arrows represented daily SDS modeling. (D) Representative image of retrograde tracing slices of CON and PS mice. Scale bar = 1 mm. (E) Quantification of whole‐brain inputs to SNc TH^+^ neurons.

Given that depression was susceptible to MPTP, we aimed to elucidate the neural mechanism of this phenomenon. Studies have revealed that the SNc receives efferent projections from other regions around the whole brain; thus, we utilized a brain‐wide viral tracing tool to detect changes in upstream inputs to SNc TH^+^ neurons. Cre‐dependent helper viruses (AAV‐DIO‐G, AAV‐DIO‐TVA‐EYFP) and AAV‐TH‐Cre were injected into the unilateral SNc on day 1. After a week of recovery, the mice were subjected to 10‐day SDS to obtain physical stress. Then, the retrograde virus RV‐EnVA‐ΔG‐dsRed was injected into the same site of the SNc. When the virus reached the peak of expression, PS mice were all sacrificed on day 28 (Figure 2A–C). Through quantification of dsRed‐positive neurons in the upstream brain regions of SNc TH^+^ cells, we found that SNc^DA^‐projecting neurons in the CeA, ZI, and SNr (CON vs. PS, CeA: *p* = 0.0004, ZI: *p* < 0.0001, SNr: *p* = 0.0032) were significantly changed in PS mice compared with CON mice (Figure 2D,E).

### 3.3 Activity of SNc^DA^‐projecting neurons in the CeA was increased in depressed mice when interacting with CD1

**FIGURE 3:**
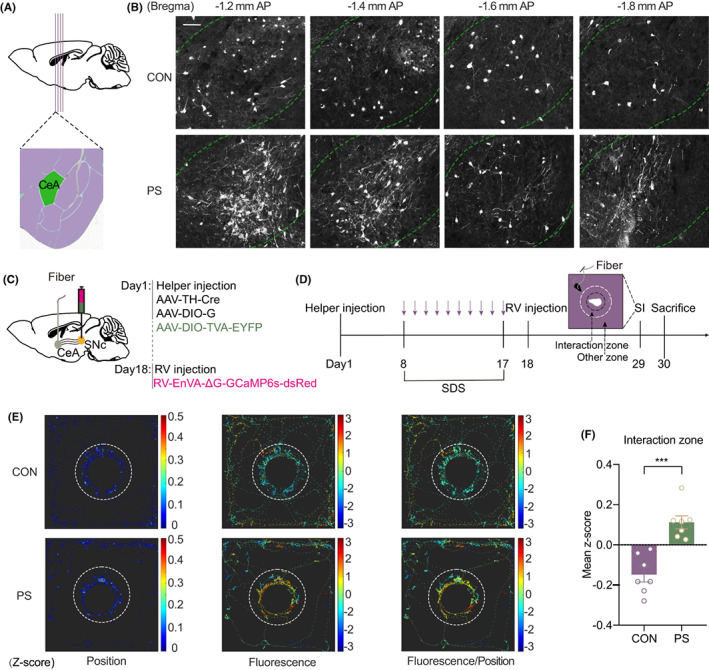
Activity of SNc^DA^‐projecting neurons in CeA was increased in depressive mice when interacting with CD1. (A) Illustration of anatomical localization of four coronal sections of target CeA. (B) Representative fluorescent images of CeA sections in (A) that project to SNc^DA^. Scale bar = 50 μm. (C) The strategy of viral injection and fiber burying. (D) Schematic representation of experiment procedure. The purple arrows represented daily SDS modeling. (E) Heat map of travel route (left), GCaMP6s signal (middle), average GCaMP6s signal at each point of travel route (right) in social interaction test. (F) Mean z‐score of GCaMP6s signal of an individual mouse in the interaction zone.

Emotional deficits are known to be associated with PD, and a key structure of emotional processing is the amygdala. In the above results, we found that projections from the CeA to the SNc^DA^ were definitely changed in PS mice (Figure 3A,B). Thus, we next detected the function of the CeA‐SNc^DA^ circuit during social interaction. To address this issue, helper viruses of AAV‐TH‐Cre, AAV‐DIO‐G, and AAV‐DIO‐TVA‐EYFP were first injected into the bilateral SNc. After 1 week of recovery, the mice were subjected to 10 days of SDS treatment. Then, RV‐EnVA‐ΔG‐GCaMP6s‐dsRed was injected into the same site of the SNc, while fibers were buried in the bilateral CeA. After the genetically encoded Ca^2+^ indicator GCaMP6s was expressed in the CeA, a social interaction test was performed on day 18 (Figure 3C,D). The dynamics of SNc^DA^‐projecting neurons in the CeA during social avoidance behaviors were recorded in real time (Figure 3E). By analysis of average Ca^2+^ activity in every position of the travel route, we found that the activity of SNc^DA^‐projecting neurons in the CeA was increased (CON vs. PS, *p* = 0.0002) in depressed mice when the aggressive CD1 mouse entered the interaction zone (Figure 3F).

### 3.4 Activating the CeA‐SNc^DA^ circuit mimicked the stress‐induced vulnerability to MPTP

**FIGURE 4:**
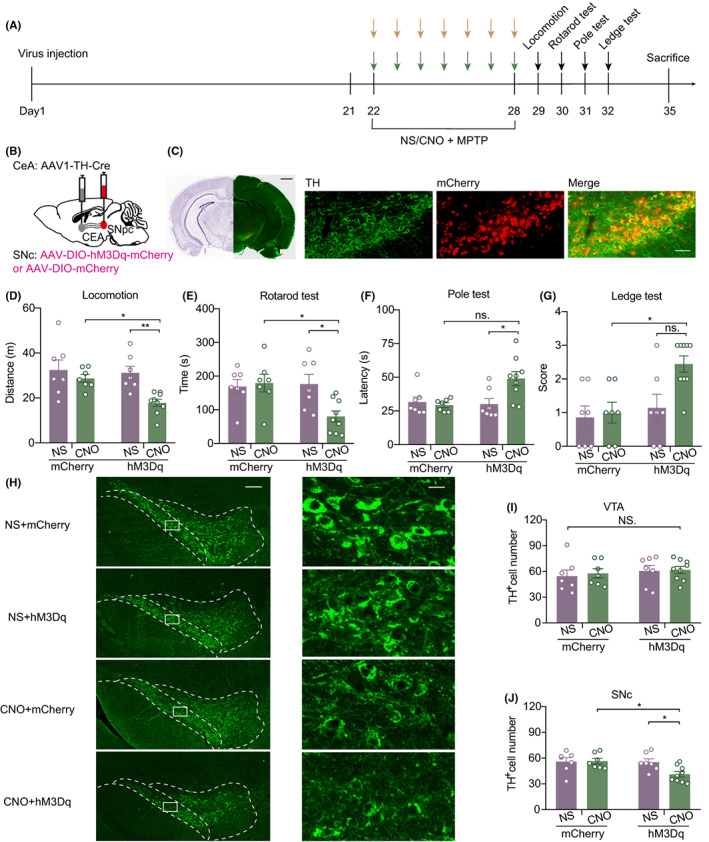
Chemogenetic activating CeA‐SNc^DA^ circuit mimicked vulnerability to MPTP as physical stress. (A) Experiment scheme for excitatory regulation of CeA‐SNc^DA^ circuit. The yellow arrows represent CNO or saline injection and the green arrows represent MPTP injection. (B) The strategy of viral injection. (C) Representative fluorescence images of virus injection position and immunofluorescence staining of TH^+^ neuron in SNc. Scale bar = 1 mm (left, black); scale bar = 50 μm (right, white). (D‐J) Behavioral evaluation and neural death induced by MPTP in individual groups. (D) Distance of mice in locomotion test. (E) Latency time of mice fall from rotarod. (F) Latency for mice get from the top to the bottom in the pole test. (G) The score of mice performance in ledge test. (H) Representative images of TH^+^ neurons immunofluorescence staining in VTA and SNc at low and high magnification. Scale bar = 150/15 μm. (I) Calculation of TH^+^ cell number in VTA. (J) The number of TH^+^ cells in SNc.

Based on the response of SNc^DA^‐projecting neurons in the CeA of PS mice during interaction with CD1, we further regulated the CeA‐SNc circuit to confirm its function in the MPTP model. Since the CeA is primarily composed of GABAergic interneurons,^26^, ^27^ we aimed to determine whether activation of the CeA‐SNc^DA^ circuit also led to susceptibility to MPTP. First, we injected an antegrade monosynaptic transneuronal AAV1 vector that expresses TH‐Cre into the bilateral CeA. Additionally, a Cre‐dependent AAV9 that expresses the excitatory hM3Dq designer receptor exclusively activated by the designer drug CNO (DREADD)^28^ was injected into the bilateral SNc (Figure 4B,C). After 3 weeks, the expression of the virus peaked. Then, the mice received MPTP injection 30 min after CNO or saline intraperitoneal injection for 7 consecutive days. One day after the last dose, a series of behavioral tests were administered. Finally, the mice were sacrificed on day 35 to detect TH^+^ neurons (Figure 4A). The results showed that the locomotion (mCherry + CNO vs. hM3Dq + CNO, *p* = 0.0421; hMsDq+NS vs. hM3Dq + CNO, *p* = 0.0092) of mice injected with hM3Dq and CNO was obviously decreased (Figure 4D). The latency to fall from the rotarod (mCherry + CNO vs. hM3Dq + CNO, *p* = 0.0199; hMsDq+NS vs. hM3Dq + CNO, *p* = 0.0252) was also shortened in mice in the hM3Dq + CNO group (Figure 4E). In the pole test (hM3Dq + NS vs. hM3Dq + CNO, *p* = 0.020) and ledge tests (mCherry + CNO vs. hM3Dq + CNO, *p* = 0.050), mice that received both hM3Dq and CNO operation received higher scores (Figure 4F,G). We observed that activating CeA‐SNc^DA^ circuits could amplify dyskinesia induced by MPTP injection. Moreover, by brain tissue immunofluorescence staining, we found that the loss of TH^+^ cells was increased in the SNc (mCherry + CNO vs. hM3Dq + CNO, *p* = 0.0276; hM3Dq + NS vs. hM3Dq + CNO, *p* = 0.0456) of hM3Dq + CNO mice but not in the VTA (Figure 4H–J). These results suggested that activating CeA‐SNc^DA^ circuits mimicked the vulnerability to MPTP as physical stress.

### 3.5 Inhibition of the CeA‐SNc^DA^ circuit reversed the MPTP vulnerability caused by depression

**FIGURE 5:**
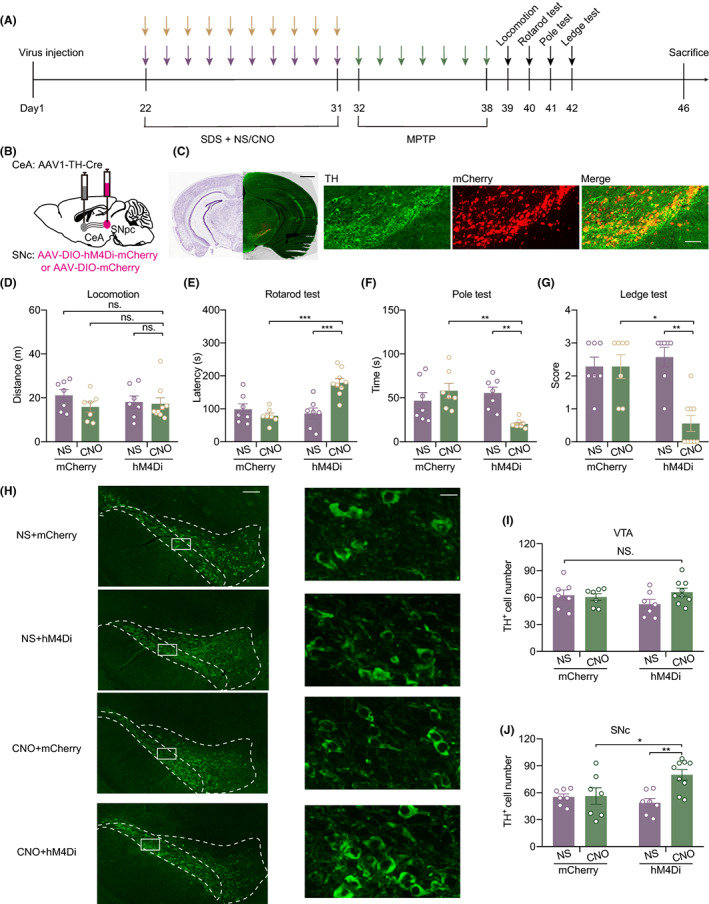
Chemogenetic inhibition of CeA‐SNc^DA^ circuit reversed the vulnerability to MPTP caused by physical stress. (A) Experiment scheme for inhibitory regulation of CeA‐SNc^DA^ circuit. The purple arrows represent depression modeling through SDS; the yellow arrows represent CNO or saline injection; and the green arrows represent MPTP injection. (B) The strategy of viral injection. (C) Representative images of virus injection position and immunofluorescence staining of TH+ neuron in SNc. Scale bar = 1 mm (left, black); scale bar = 50 μm (right, white). (D–J) Behavioral evaluation and neural death induced by SDS and MPTP in individual groups. (D) Distance of mice in Locomotion test. (E) Latency time of mice to fall from rotarod. (F) Latency for mice to get from the top to the bottom in the pole test. (G) The score of mice performance in ledge test. (H) Representative images of TH^+^ neurons immunofluorescence staining in VTA and SNc at low and high magnification. Scale bar = 150/15 μm. (I) Calculation of TH^+^ cell number in VTA. (J) The number of TH^+^ cells in SNc.

To determine whether the CeA‐SNc^DA^ circuit is required for depression‐aggravating damage caused by MPTP, we injected inhibitory AAV‐DIO‐hM4Di‐mCherry or AAV‐DIO‐mCherry as a control virus into the SNc, while AAV1‐TH‐Cre was injected into the CeA (Figure 5A–C). After viral expression, mice received 10 days of SDS to establish a depressive‐like model and CNO/saline injection daily. Then, all mice were subjected to MPTP treatment for a week. Next, the same series of behavioral tests were carried out. Finally, the mice were sacrificed to detect TH^+^ cells (Figure 5A). According to the results of behavioral tests, we found that the distance traveled in the locomotion test was not different among all mice (Figure 5D), but mice injected with both hM4Di and CNO expressed more latency time (mCherry + CNO vs. hM4Di + CNO, *p* = 0.0001; hM4Di + NS vs. hM4Di + CNO, *p* = 0.0003) to fall off the rotarod than the other groups (Figure 5E). This result implied that inhibition of the CeA‐SNc^DA^ circuit is beneficial for improving the ability but not the willingness of movement in mice. In the pole test (mCherry + CNO vs. hM4Di + CNO, *p* = 0.0020; hM4Di + NS vs. hM4Di + CNO, *p* = 0.0040) and ledge test (mCherry + CNO vs. hM4Di + CNO, *p* = 0.021; hM4Di + NS vs. hM4Di + CNO, *p* = 0.003), mice in the hM4Di + CNO group displayed better performance than the other groups (Figure 5F,G). Through brain tissue immunofluorescence staining (Figure 5H), we found that the number of TH^+^ cells in the VTA was not different among the four groups (Figure 5I). This phenomenon suggested that the VTA is not an impressionable target of MPTP. However, SNc showed a greater number of TH^+^ cells (mCherry + CNO vs. hM4Di + CNO, *p* = 0.0473; hM4Di + NS vs. hM4Di + CNO, *p* = 0.0059) in hM4Di + CNO mice (Figure 5J), which means inhibition of the CeA‐SNc^DA^ circuit rescued SNc DA neuron loss. Taken together, these results revealed that inhibition of the CeA‐SNc^DA^ circuit could reverse the susceptibility to MPTP caused by physical stress.

## 4 DISCUSSION

In this exploratory study, we investigated whether stress‐induced mood disorders contribute to sensitivity to the parkinsonian toxin MPTP and which neural circuit was involved in this process. There are three main findings. First, mice with PS, but not ES, were more susceptible to MPTP with worse dyskinesia and more DA neuron death in the SNc (Figure 1). Furthermore, the projection from the CeA to SNc^DA^ was increased (Figure 2), and the activity of this pathway was enhanced in mice with PS during interaction with aggressive CD1 mice (Figure 3). In line with these results, we also found that activating the CeA‐SNc^DA^ circuit could mimic the vulnerability to MPTP (Figure 4) and that inhibiting this pathway could reverse vulnerability to MPTP caused by PS (Figure 5).

Notably, in this study, we found that PS but not ES led to susceptibility to MPTP, although anxiety disorder also commonly occurred in PD as one of the nonmotor symptoms.^29^ Previous studies have shown that MPTP‐treated mice reproduce the features of anxiety,^30^ and it was reported that MPTP‐induced dopamine depletion in the basolateral amygdala led to anxiety‐like behaviors.^31^ However, studies on the influence of anxiety on PD are relatively rare. In our study, mice with ES showed neuropathology caused by MPTP similar to that of control mice. Moreover, ES mice with MPTP treatment showed no significant change in motor ability or neuronal death compared with ES mice without MPTP administration. This result indicated that ES‐induced anxiety may not lead to the vulnerability of DA neurons to the parkinsonian toxin MPTP. The different impact between PS and ES on the movement performance and SNc DA neurons death may be attributed to two aspects. Firstly, the PS mice had worse performance and more neuron death than ES mice, which may be ascribed to the PS group undergoing severer stress than ES in the three‐chamber SDS modeling. A previously published study has also suggested that ES‐exposed mice, compared with the PS‐exposed group, exhibited a much lesser degree of social avoidant behavior.^32^ Secondly, the structural connectivity of the SNc DA neurons may be also involved in the difference between these groups. Along with the whole brain mapping of the direct inputs of SNc DA neurons in PS mice, the number of labeled CeA neurons in the PS group was approximately twice as many as that in controls (Figure 2E). Undoubtedly, the comparison of brain structural changes between ES mice and control mice is necessary for clarifying the reason why ES mice have no influence on the reactivity of SNc DA neurons to MPTP. Thus, this result and the potential mechanism remain to be further researched, which is a limitation of this study.

The CeA is a critical nucleus of the striatum involved in aversive stress processing.^33^ For example, during fear conditioning, conditioned stimulus and unconditioned stimulus are relayed to the lateral nucleus of the amygdala from thalamic and cortical regions of auditory and somatosensory systems, respectively.^34^, ^35^, ^36^ Conditioned stimulus information is then transmitted through the lateral amygdala and surrounding nuclei to the CeA to mediate fear conditioning. Unconditioned stimulus inputs to the CeA could be involved in higher‐order integration. In addition, the CeA projects to brainstem areas that control the expression of stress responses. For example, damage to the lateral hypothalamus affects blood pressure, damage to periaqueductal gray interferes with freezing,^37^ and damage to the bed nucleus disrupts the conditioned release of pituitary–adrenal stress hormones.^38^ In our study, we extended the current knowledge of the function of the CeA and revealed enhanced activity of CeA in promoting the advance of PD.

In addition to the CeA, we also detected apparent changes in projections from SNr and ZI to SNc DA neurons. Whether these two nuclei were related to susceptibility to MPTP remained unclear in this study. It was reported that ZI plays a role in the initiation and control of movement,^39^ but near‐infrared light treatment protected SNc DA neurons instead of ZI from MPTP.^40^ SNr, a major basal ganglia output nucleus, plays a vital role in movement execution.^41^ It was reported that SNr displayed abnormalities in neural discharge caused by MPTP.^42^ The dopamine transporter and TH in the mouse SNr were also reduced due to MPTP.^43^ In addition, studies have revealed that the SNr‐thalamus projections support the continuity of motor patterns, while SNr–SNc projections modulate the immediate motor drive behind them.^44^

CNO, the most widely used synthetic DREADD ligand, is able to back metabolized to clozapine (CLZ). CLZ accumulated in brain tissue can occupy dopamine and serotonin 5HT receptors in the living brain. It is unknown Whether CLZ has DREADD‐independent effects on behavior. In our study, CNO were delivered 3 mg/kg daily and behavioral tests were measured more than 24 h after the last dose. It was reported that CNO itself can enter the brain, and free cerebrospinal fluid levels were within the range to activate DREADD directly, while CLZ level remained below the detection limit, and the half maximal effective concentration (EC50) of CLZ was substantially lower (0.42 nM) than CNO (8.1 nM).^45^ Furthermore, there was a study finding a CNO dose‐dependent effect on the availability of both neuroreceptor sites,^46^ and it was 5, but not 3 mg/kg, a dose of CNO reduced cocaine‐induced locomotion.^47^ For the chronic applications, non‐DREADD‐expressing mice that received i.p. vehicle, CNO, or compound 21 (C21) 5 days/week for 16 weeks showed largely lack of behavioral effects.^48^ Thus, studies utilizing DREADDs to probe behaviors should consider this limitation when choosing a dose of CNO and include non‐DREADD CNO controls.

The current treatments for PD are involved in various molecular targets, including α‐synuclein, transcription factor EB,^49^ striatal metabotropic glutamate receptor type 5, and autophagic pathways.^50^ Meanwhile, the mucuna pruriens,^51^ ursolic acid,^52^, ^53^, ^54^ and chlorogenic acid^55^, ^56^ also have the neuroprotective effect on DA neurons in PD animal models. In this study, our findings demonstrate that depression promotes MPTP‐induced features and that the CeA‐SNc^DA^ pathway plays a critical role in this process. Further studies applying neural circuit intervention therapy may be applied in patients to prevent and mitigate dyskinesia and neurodegeneration.

## AUTHOR CONTRIBUTION

Hongwei Cai and Pei Zhang performed animal behavior studies. Jian Yang and Kun Ren established the animal model. Hongwei Cai, Tongxia Li, and Jie Lei performed virus proportioning and injection. Ming Li, Lijun Zhang, and Chi Cui performed fluorescence experiments. Hongwei Cai and Pei Zhang performed statistical analysis of the data and wrote the manuscript. Jie Ming and Bo Tian designed the research and revised the paper. Jie Ming and Bo Tian supervised the study.

## FUNDING INFORMATION

This work was supported by grants from National Natural Science Foundation of China (Nos. 31871073 and 32171023 to B.T. and No. 32271036 to P.Z.). All authors report no conflicts of interest.

## CONFLICT OF INTEREST STATEMENT

The authors declare no competing interests.

## Supporting information


Table S1.

Appendix S1.

## Data Availability

The data that support the findings of this study are available from the corresponding author upon reasonable request.

## References

[1] Jankovic J . Parkinson's disease: clinical features and diagnosis. J Neurol Neurosurg Psychiatry. 2008;79(4):368‐376. 18344392 10.1136/jnnp.2007.131045

[2] Marinus J , Zhu K , Marras C , Aarsland D , van Hilten JJ . Risk factors for non‐motor symptoms in Parkinson's disease. Lancet Neurol. 2018;17(6):559‐568. 29699914 10.1016/S1474-4422(18)30127-3

[3] Rai SN , Singh P . Advancement in the modelling and therapeutics of Parkinson's disease. J Chem Neuroanat. 2020;104:101752. 31996329 10.1016/j.jchemneu.2020.101752

[4] Helmich RC , Bloem BR . The impact of the COVID‐19 pandemic on Parkinson's disease: hidden sorrows and emerging opportunities. J Parkinsons Dis. 2020;10(2):351‐354. 32250324 10.3233/JPD-202038 PMC7242824

[5] Zou K , Guo W , Tang G , Zheng B , Zheng Z . A case of early onset Parkinson's disease after major stress. Neuropsychiatr Dis Treat. 2013;9:1067‐1069. 23950648 10.2147/NDT.S48455 PMC3742351

[6] Schwab RS , Zieper I . Effects of mood, motivation, stress and alertness on the performance in Parkinson's disease. Psychiatr Neurol (Basel). 1965;150(6):345‐357. 5865993 10.1159/000127780

[7] Rothman SM , Mattson MP . Adverse stress, hippocampal networks, and Alzheimer's disease. Neuromolecular Med. 2010;12(1):56‐70. 19943124 10.1007/s12017-009-8107-9 PMC2833224

[8] Hemmerle AM , Herman JP , Seroogy KB . Stress, depression and Parkinson's disease. Exp Neurol. 2012;233(1):79‐86. 22001159 10.1016/j.expneurol.2011.09.035 PMC3268878

[9] Smith LK , Jadavji NM , Colwell KL , Katrina Perehudoff S , Metz GA . Stress accelerates neural degeneration and exaggerates motor symptoms in a rat model of Parkinson's disease. Eur J Neurosci. 2008;27(8):2133‐2146. 18412632 10.1111/j.1460-9568.2008.06177.x PMC5222623

[10] Smith AD , Castro SL , Zigmond MJ . Stress‐induced Parkinson's disease: a working hypothesis. Physiol Behav. 2002;77(4–5):527‐531. 12526994 10.1016/s0031-9384(02)00939-3

[11] Rai SN , Tiwari N , Singh P , et al. Exploring the paradox of COVID‐19 in neurological complications with emphasis on Parkinson's and Alzheimer's disease. Oxid Med Cell Longev. 2022;2022:3012778‐3012716. 36092161 10.1155/2022/3012778 PMC9453010

[12] van der Heide A , Speckens AEM , Meinders MJ , Rosenthal LS , Bloem BR , Helmich RC . Stress and mindfulness in Parkinson's disease ‐ a survey in 5000 patients. NPJ Parkinsons Dis. 2021;7(1):7. 33462213 10.1038/s41531-020-00152-9 PMC7813889

[13] Qi G , Zhang P , Li T , et al. NAc‐VTA circuit underlies emotional stress‐induced anxiety‐like behavior in the three‐chamber vicarious social defeat stress mouse model. Nat Commun. 2022;13(1):577. 35102141 10.1038/s41467-022-28190-2 PMC8804001

[14] Obeso JA , Rodriguez‐Oroz MC , Benitez‐Temino B , et al. Functional organization of the basal ganglia: therapeutic implications for Parkinson's disease. Mov Disord. 2008;23(Suppl 3):S548‐S559. 18781672 10.1002/mds.22062

[15] Lim J , Bang Y , Choi HJ . Abnormal hippocampal neurogenesis in Parkinson's disease: relevance to a new therapeutic target for depression with Parkinson's disease. Arch Pharm Res. 2018;41(10):943‐954. 30136247 10.1007/s12272-018-1063-x

[16] Davie CA . A review of Parkinson's disease. Br Med Bull. 2008;86(1):109‐127. 18398010 10.1093/bmb/ldn013

[17] Burke RE , O'Malley K . Axon degeneration in Parkinson's disease. Exp Neurol. 2013;246:72‐83. 22285449 10.1016/j.expneurol.2012.01.011 PMC3340476

[18] Gabbay V , Ely BA , Li Q , et al. Striatum‐based circuitry of adolescent depression and anhedonia. J Am Acad Child Adolesc Psychiatry. 2013;52(6):628‐641 e613. 23702452 10.1016/j.jaac.2013.04.003 PMC3762469

[19] Kaasinen V , Vahlberg T , Stoessl AJ , Strafella AP , Antonini A . Dopamine receptors in Parkinson's disease: a meta‐analysis of imaging studies. Mov Disord. 2021;36(8):1781‐1791. 33955044 10.1002/mds.28632

[20] Chou JS , Chen CY , Chen YL , et al. (G2019S) LRRK2 causes early‐phase dysfunction of SNpc dopaminergic neurons and impairment of corticostriatal long‐term depression in the PD transgenic mouse. Neurobiol Dis. 2014;68:190‐199. 24830390 10.1016/j.nbd.2014.04.021

[21] Kim S , Kwon SH , Kam TI , et al. Transneuronal propagation of pathologic alpha‐synuclein from the gut to the brain models Parkinson's disease. Neuron. 2019;103(4):627‐641 e627. 31255487 10.1016/j.neuron.2019.05.035 PMC6706297

[22] Trnka R , Hasto J , Cabelkova I , Kuska M , Tavel P , Nikolai T . Amygdala and emotionality in Parkinson's disease: an integrative review of the neuropsychological evidence. Neuro Endocrinol Lett. 2018;39(2):105‐110. 29919988

[23] Li B , Ge T , Cui R . Long‐term plasticity in amygdala circuits: implication of CB1‐dependent LTD in stress. Mol Neurobiol. 2018;55(5):4107‐4114. 28593436 10.1007/s12035-017-0643-y

[24] Golden SA , Covington HE 3rd , Berton O , Russo SJ . A standardized protocol for repeated social defeat stress in mice. Nat Protoc. 2011;6(8):1183‐1191. 21799487 10.1038/nprot.2011.361 PMC3220278

[25] Zhang QS , Heng Y , Mou Z , Huang JY , Yuan YH , Chen NH . Reassessment of subacute MPTP‐treated mice as animal model of Parkinson's disease. Acta Pharmacol Sin. 2017;38(10):1317‐1328. 28649132 10.1038/aps.2017.49 PMC5630672

[26] Gilpin NW , Herman MA , Roberto M . The central amygdala as an integrative hub for anxiety and alcohol use disorders. Biol Psychiatry. 2015;77(10):859‐869. 25433901 10.1016/j.biopsych.2014.09.008 PMC4398579

[27] Hardaway JA , Halladay LR , Mazzone CM , et al. Central amygdala Prepronociceptin‐expressing neurons mediate palatable food consumption and reward. Neuron. 2019;102(5):1037‐1052. 31029403 10.1016/j.neuron.2019.03.037 PMC6750705

[28] Armbruster BN , Li X , Pausch MH , Herlitze S , Roth BL . Evolving the lock to fit the key to create a family of G protein‐coupled receptors potently activated by an inert ligand. P Natl Acad Sci USA. 2007;104(12):5163‐5168. 10.1073/pnas.0700293104 PMC1829280 17360345

[29] Khatri DK , Choudhary M , Sood A , Singh SB . Anxiety: an ignored aspect of Parkinson's disease lacking attention. Biomed Pharmacother. 2020;131:110776. 33152935 10.1016/j.biopha.2020.110776

[30] Han NR , Kim YK , Ahn S , Hwang TY , Lee H , Park HJ . A comprehensive phenotype of non‐motor impairments and distribution of alpha‐synuclein deposition in parkinsonism‐induced mice by a combination injection of MPTP and probenecid. Front Aging Neurosci. 2021;12:12. 10.3389/fnagi.2020.599045 PMC7838388 33519420

[31] Zhang TT , Chen TT , Chen PP , Zhang BF , Hong J , Chen L . MPTP‐induced dopamine depletion in basolateral amygdala via decrease of D2R activation suppresses GABA(a) receptors expression and LTD induction leading to anxiety‐like behaviors. Front Mol Neurosci. 2017;10:1‐15. 28824377 10.3389/fnmol.2017.00247 PMC5545577

[32] Warren BL , Vialou VF , Iniguez SD , et al. Neurobiological sequelae of witnessing stressful events in adult mice. Biol Psychiatry. 2013;73(1):7‐14. 22795644 10.1016/j.biopsych.2012.06.006 PMC3498570

[33] Medina JF , Repa JC , Mauk MD , LeDoux JE . Parallels between cerebellum and amygdala‐dependent conditioning. Nat Rev Neurosci. 2002;3(2):122‐131. 11836520 10.1038/nrn728

[34] McDonald AJ . Cortical pathways to the mammalian amygdala. Prog Neurobiol. 1998;55(3):257‐332. 9643556 10.1016/s0301-0082(98)00003-3

[35] Mascagni F , McDonald AJ , Coleman JR . Corticoamygdaloid and corticocortical projections of the rat temporal cortex: a Phaseolus vulgaris leucoagglutinin study. Neuroscience. 1993;57(3):697‐715. 8309532 10.1016/0306-4522(93)90016-9

[36] Romanski LM , LeDoux JE . Information cascade from primary auditory cortex to the amygdala: corticocortical and corticoamygdaloid projections of temporal cortex in the rat. Cereb Cortex. 1993;3(6):515‐532. 7511012 10.1093/cercor/3.6.515

[37] LeDoux JE , Iwata J , Cicchetti P , Reis DJ . Different projections of the central amygdaloid nucleus mediate autonomic and behavioral correlates of conditioned fear. J Neurosci. 1988;8(7):2517‐2529. 2854842 10.1523/JNEUROSCI.08-07-02517.1988 PMC6569498

[38] Van de Kar LD , Piechowski RA , Rittenhouse PA , Gray TS . Amygdaloid lesions: differential effect on conditioned stress and immobilization‐induced increases in corticosterone and renin secretion. Neuroendocrinology. 1991;54(2):89‐95. 1766554 10.1159/000125856

[39] Nandi D , Aziz TZ , Liu X , Stein JF . Brainstem motor loops in the control of movement. Mov Disord. 2002;17(Suppl 3):S22‐S27. 11948752 10.1002/mds.10139

[40] Shaw VE , Spana S , Ashkan K , et al. Neuroprotection of midbrain dopaminergic cells in MPTP‐treated mice after near‐infrared light treatment. J Comp Neurol. 2010;518(1):25‐40. 19882716 10.1002/cne.22207

[41] Faynveitz A , Lavian H , Jacob A , Korngreen A . Proliferation of inhibitory input to the substantia Nigra in experimental parkinsonism. Front Cell Neurosci. 2019;13:13. 31572130 10.3389/fncel.2019.00417 PMC6753199

[42] Wichmann T , Bergman H , Starr PA , Subramanian T , Watts RL , DeLong MR . Comparison of MPTP‐induced changes in spontaneous neuronal discharge in the internal pallidal segment and in the substantia nigra pars reticulata in primates. Exp Brain Res. 1999;125(4):397‐409. 10323285 10.1007/s002210050696

[43] Fujita A , Fujita Y , Pu YY , Chang LJ , Hashimoto K . MPTP‐induced dopaminergic neurotoxicity in mouse brain is attenuated after subsequent intranasal administration of (R)‐ketamine: a role of TrkB signaling. Psychopharmacology (Berl). 2020;237(1):83‐92. 31418048 10.1007/s00213-019-05346-5

[44] Rizzi G , Tan KR . Synergistic nigral output pathways shape movement. Cell Rep. 2019;27(7):2184‐+:2184‐2198.e4. 31091455 10.1016/j.celrep.2019.04.068

[45] Jendryka M , Palchaudhuri M , Ursu D , et al. Pharmacokinetic and pharmacodynamic actions of clozapine‐N‐oxide, clozapine, and compound 21 in DREADD‐based chemogenetics in mice. Sci Rep. 2019;9(1):4522. 30872749 10.1038/s41598-019-41088-2 PMC6418145

[46] Baerentzen S , Casado‐Sainz A , Lange D , et al. The Chemogenetic receptor ligand clozapine N‐oxide induces in vivo neuroreceptor occupancy and reduces striatal glutamate levels. Front Neurosci. 2019;13:187. 31001069 10.3389/fnins.2019.00187 PMC6456655

[47] Padovan‐Hernandez Y , Knackstedt LA . Dose‐dependent reduction in cocaine‐induced locomotion by clozapine‐N‐oxide in rats with a history of cocaine self‐administration. Neurosci Lett. 2018;674:132‐135. 29571824 10.1016/j.neulet.2018.03.045 PMC5899660

[48] Tran FH , Spears SL , Ahn KJ , Eisch AJ , Yun S . Does chronic systemic injection of the DREADD agonists clozapine‐N‐oxide or compound 21 change behavior relevant to locomotion, exploration, anxiety, and depression in male non‐DREADD‐expressing mice? Neurosci Lett. 2020;739:135432. 33080350 10.1016/j.neulet.2020.135432

[49] Rai SN , Tiwari N , Singh P , et al. Therapeutic potential of vital transcription factors in Alzheimer's and Parkinson's disease with particular emphasis on transcription factor EB mediated autophagy. Front Neurosci. 2021;15:777347. 34970114 10.3389/fnins.2021.777347 PMC8712758

[50] Rai SN , Singh P , Varshney R , et al. Promising drug targets and associated therapeutic interventions in Parkinson's disease. Neural Regen Res. 2021;16(9):1730‐1739. 33510062 10.4103/1673-5374.306066 PMC8328771

[51] Rai SN , Chaturvedi VK , Singh P , Singh BK , Singh MP . Mucuna pruriens in Parkinson's and in some other diseases: recent advancement and future prospective. 3 Biotech. 2020;10(12):522. 10.1007/s13205-020-02532-7 PMC7655893 33194526

[52] Zahra W , Rai SN , Birla H , et al. Neuroprotection of rotenone‐induced parkinsonism by Ursolic acid in PD mouse model. CNS Neurol Disord Drug Targets. 2020;19(7):527‐540. 32787765 10.2174/1871527319666200812224457

[53] Rai SN , Zahra W , Singh SS , et al. Anti‐inflammatory activity of Ursolic acid in MPTP‐induced parkinsonian mouse model. Neurotox Res. 2019;36(3):452‐462. 31016688 10.1007/s12640-019-00038-6

[54] Rai SN , Yadav SK , Singh D , Singh SP . Ursolic acid attenuates oxidative stress in nigrostriatal tissue and improves neurobehavioral activity in MPTP‐induced parkinsonian mouse model. J Chem Neuroanat. 2016;71:41‐49. 26686287 10.1016/j.jchemneu.2015.12.002

[55] Singh SS , Rai SN , Birla H , et al. Neuroprotective effect of chlorogenic acid on mitochondrial dysfunction‐mediated apoptotic death of DA neurons in a parkinsonian mouse model. Oxid Med Cell Longev. 2020;2020:6571484 14. 32566093 10.1155/2020/6571484 PMC7273475

[56] Singh SS , Rai SN , Birla H , et al. Effect of chlorogenic acid supplementation in MPTP‐intoxicated mouse. Front Pharmacol. 2018;9:757. 30127737 10.3389/fphar.2018.00757 PMC6087758

