# Supplementary material for: Amygdalo‐nigral circuit mediates stress‐induced vulnerability to the parkinsonian toxin MPTP
Source: CNS Neurosci Ther. 2023 Mar 13;29(7):1940–52. doi: 10.1111/cns.14151 (PMC10324352; doi:10.1111/cns.14151)
Supplement: Supplementary file 1 — Table S1. [file CNS-29-1940-s001.pdf]

Statistical Report

figure number	n	Statistic method	P value	F/t value	Post hoc multiple compatisons test
Figure 1B	CON, n=20	Normality test_Shapiro-Wilk (W)	p=0.4806	0.9567	\
	PS, n=16		p=0.5324	0.9526	\
	ES, n=16		p=0.7899	0.9671	\
	CON, n=20; PS, n=16; ES, n=16.	Ordinary one-way ANOVA, Dunnett's multiple comparisons test.	p=0.0018	F(2,49)=7.226	CON vs. PS, p=0.0010**; CON vs. ES, p=0.0432*.
Figure 1C	CON, n=20	Normality test_Shapiro-Wilk (W)	p=0.0153	0.8765	\
	PS, n=16		p=0.0795	0.8997	\
	ES, n=16		p=0.1358	0.9142	\
	CON, n=20	Nonparametric test	CON vs. ES, p=0.406	\	\
	PS, n=16		CON vs. PS, p=0.004*	\	\
	ES, n=16		PS vs. ES, p=0.301	\	\
Figure 1D	CON, n=20	Normality test_Shapiro-Wilk (W)	p=0.3417	0.9483	\
	PS, n=16		p=0.0104	0.842	\
	ES, n=16		p=0.1444	0.9158	\
	CON, n=20	Nonparametric test	CON vs. ES, p=0.014	\	\
	PS, n=16		CON vs. PS, p=0.001**	\	\
	ES, n=16		PS vs. ES, p=0.585	\	\
Figure 1E	CON, n=20	Normality test_Shapiro-Wilk (W)	p=0.048	0.9035	\
	PS, n=16		p=0.0109	0.8434	\
	ES, n=16		p=0.0028	0.8006	\
	CON, n=20	Nonparametric test	CON vs. ES, p=0.060	\	\
	PS, n=16		CON vs. PS, p=0.019*	\	\
	ES, n=16		PS vs. ES, p=1.000	\	\
Figure 1F	CON, n=20	Normality test_Shapiro-Wilk (W)	p=0.0006	0.7871	\
	PS, n=16		p=0.007	9.927	\
	ES, n=16		p=0.0017	0.7845	\
	CON, n=20	Nonparametric test	CON vs. ES, p=1.000	\	\
	PS, n=16		CON vs. PS, p=0.000076****	\	\
	ES, n=16		PS vs. ES, p=0.001	\	\
	CON+NS, n=10	Normality test_Shapiro-Wilk (W)	p=0.7286	0.9551	\
	PS+NS, n=8;		p=0.2237	0.8879	\
	ES+NS, n=8;		p=0.1751	0.8767	\
	CON+MPTP, n=10;		p=0.388	0.9236	\
	PS+MPTP, n=8;		p=0.149	0.8695	\
	ES+MPTP, n=8.		p=0.6849	0.9474	\

Figure 1G	CON+NS, n=10;<br>PS+NS, n=8;<br>ES+NS, n=8;<br>CON+MPTP, n=10;<br>PS+MPTP, n=8;<br>ES+MPTP, n=8.	Two-way ANOVA,<br>Tukey's multiple comparison test.	\	\	Interaction:F(2,46)=1.143,P=0.3277<br>Treatment:F(2,46)=7.616,P=0.0014<br>Group:F(1,46)=45.16,P<0.0001<br>multiple comparisons:<br>CON+NS vs. PS+NS, p = 0.0441*;<br>CON+NS vs. CON+MPTP, p < 0.0001****;<br>PS+NS vs. PS+MPTP, p = 0.0080**.
Figure 1H	CON+NS, n=10	Normality test_Shapiro-Wilk (W)	p=0.4726	0.9325	\
	PS+NS, n=8;		p=0.5	0.9282	\
	ES+NS, n=8;		p=0.5399	0.9326	\
	CON+MPTP, n=10;		p=0.0779	0.8607	\
	PS+MPTP, n=8;		p=0.8986	0.9701	\
	ES+MPTP, n=8.		p=0.3189	0.9048	\
	CON+NS, n=10;<br>PS+NS, n=8;<br>ES+NS, n=8;<br>CON+MPTP, n=10;<br>PS+MPTP, n=8;<br>ES+MPTP, n=8.	Two-way ANOVA,<br>Tukey's multiple comparison test.	\	\	Interaction:F(2,46)=0.7063,P=0.4987<br>Treatment:F(2,46)=7.702,P=0.0013<br>Group:F(1,46)=27.23,P<0.0001<br>multiple comparisons:<br>CON+NS vs. CON+MPTP, p=0.0330*;<br>PS+NS vs. PS+MPTP, p=0.0053**;<br>CON+MPTP vs. PS+MPTP, p=0.0483*.
Figure 1I	CON+NS, n=10	Normality test_Shapiro-Wilk (W)	p=0.2466	0.9047	\
	PS+NS, n=8;		p=0.1117	0.8569	\
	ES+NS, n=8;		p=0.2355	0.8903	\
	CON+MPTP, n=10;		p=0.1276	0.8792	\
	PS+MPTP, n=8;		p=0.4814	0.9261	\
	ES+MPTP, n=8.		p=0.1269	0.8624	\
	CON+NS, n=10;<br>PS+NS, n=8;<br>ES+NS, n=8;<br>CON+MPTP, n=10;<br>PS+MPTP, n=8;<br>ES+MPTP, n=8.	Two-way ANOVA,<br>Tukey's multiple comparison test.	\	\	Interaction:F(2,46)=0.9839, P=0.3816<br>Treatment:F(2,46)=8.237, P=0.0009<br>Group:F(1,46)=23.88, P<0.0001<br>multiple comparisons:<br>CON+NS vs. CON+MPTP, p=0.0136*;<br>PS+NS vs. PS+MPTP, p=0.0140*;<br>CON+MPTP vs. PS+MPTP, p=0.0434*.
Figure 1J	CON+NS, n=10	Normality test_Shapiro-Wilk (W)	p<0.0001	0.3657	\
	PS+NS, n=8;		p=0.0555	0.8272	\
	ES+NS, n=8;		p=0.0356	0.8089	\
	CON+MPTP, n=10;		p=0.0123	0.7941	\
	PS+MPTP, n=8;		p=0.0672	0.8352	\
	ES+MPTP, n=8.		p=0.4082	0.9173	\
	CON+NS, n=10	Nonparametric test	CON+NS vs.PS+NS, p=1.000	\	\
	PS+NS, n=8;		CON+NS vs.ES+NS, p=1.000	\	\
	ES+NS, n=8;		CON+NS vs.CON+MPTP, p=0.047*	\	\
	CON+MPTP, n=10;		PS+NS vs. PS+MPTP, p=0.152	\	\
	PS+MPTP, n=8;		CON+MPTP vs. PS+MPTP, p=1.000	\	\
	ES+MPTP, n=8.		CON+MPTP vs. ES+MPTP, p=1.000	\	\
	CON+NS, n=10		p=0.8045	0.9616	\
	PS+NS, n=8;		p=0.9808	0.9843	\

Figure 1L	ES+NS, n=8;		Normality test_Shapiro-Wilk (W)	p=0.5102	0.9294	\
	CON+MPTP, n=10;			p=0.5702	0.9415	\
	PS+MPTP, n=8;			p=0.7042	0.9493	\
	ES+MPTP, n=8.			p=0.8649	0.966	\
	CON+NS, n=10;<br>PS+NS, n=8;<br>ES+NS, n=8;<br>CON+MPTP, n=10;<br>PS+MPTP, n=8;<br>ES+MPTP, n=8.		Two-way ANOVA,<br>Tukey's multiple comparison test.	\	\	Interaction:F(2,46)=0.5094, P=0.6068<br>Treatment:F(2,46)=3.425, P=0.0411<br>Group:F(1,46)=2.549, P=0.1172<br>multiple comparisons:<br>CON+NS vs. CON+MPTP, p=0.9992;<br>PS+NS vs. PS+MPTP, p=0.9795;<br>CON+MPTP vs. PS+MPTP, p=0.3794.
Figure 1M	CON+NS, n=10		Normality test_Shapiro-Wilk (W)	p=0.5115	0.9362	\
	PS+NS, n=8;			p=0.8673	0.9663	\
	ES+NS, n=8;			p=0.9399	0.9759	\
	CON+MPTP, n=10;			p=0.6364	0.9473	\
	PS+MPTP, n=8;			p=0.5167	0.9301	\
	ES+MPTP, n=8.			p=0.9695	0.9814	\
	CON+NS, n=10;<br>PS+NS, n=8;<br>ES+NS, n=8;<br>CON+MPTP, n=10;<br>PS+MPTP, n=8;<br>ES+MPTP, n=8.		Two-way ANOVA,<br>Tukey's multiple comparison test.	\	\	Interaction:F(2,46)=0.5679, P=0.5706<br>Treatment:F(2,46)=8.846, P=0.0006<br>Group:F(1,46)=23.42, P<0.0001<br>multiple comparisons:<br>CON+NS vs. CON+MPTP, p=0.0419*;<br>PS+NS vs. PS+MPTP, p=0.0148*;<br>CON+MPTP vs. PS+MPTP, p = 0.0401*.
Figure 2E	CON, n=5;<br>PS, n=4.	MOp	Normality test_Shapiro-Wilk (W)	CON: p=0.0713	0.7932	\
				PS: p=0.5174	0.9165	\
		ACB		CON: p=0.8454	0.9655	\
				PS: p=0.6685	0.9423	\
		CP		CON: p=0.1074	0.8153	\
				PS: p=0.986	0.996	\
		SI		CON: p=0.3308	0.8846	\
				PS: p=0.1797	0.8344	\
		Gpe		CON: p=0.7952	0.9582	\
				PS: p=0.1551	0.825	\
		CeA		CON: p=0.6611	0.9393	\
				PS: p=0.1832	0.8357	\
		PVH		CON: p=0.8657	0.9685	\
				PS: p=0.1887	0.8377	\
		ZI		CON: p=0.4363	0.9047	\
				PS: p=0.7981	0.9631	\
		SNr		CON: p=0.1938	0.8498	\
				PS: p=0.2281	0.8506	\
		PAG		CON: p=0.1207	0.8219	\
				PS: p=0.8999	0.9796	\
		SCm		CON: p=0.378	0.8941	\
				PS: p=0.3232	0.8763	\
		IC		CON: p=0.1611	0.8386	\

		IC		PS: p=0.8623	0.9734	\
		PB		CON: p=0.466	0.9097	\
				PS: p=0.8905	0.978	\
		IP		CON: p=0.715	0.9469	\
				PS: p=0.2059	0.8435	\
		MOp	two-tailed unpaired t test	p=0.3957;	\	\
		ACB		p=0.2285;		
		CP		p=0.9070;		
		SI		p=0.2423;		
		Gpe		p=0.1757;		
		CeA		p=0.0004***;		
		PVH		p=0.6683;		
		ZI		p<0.0001****;		
		SNr		p=0.0032**;		
		PAG		p=0.4583;		
		SCm		p=0.0943;		
		IC		p=0.5092;		
		PB		p=0.7924;		
		IP		p=0.1762.		
Figure 3F	CON, n=7;		Normality test_Shapiro-Wilk (W)	p=0.6347	0.9396	\
	PS, n=7.			p=0.1175	0.8479	\
	CON, n=7;<br>PS, n=7.		two-tailed unpaired t test	P=0.0002***	\	\
Figure 4D	mCherry+NS, n=7		Normality test_Shapiro-Wilk (W)	p=0.652	0.9415	\
	mCherry+CNO, n=7			p=0.6123	0.9371	\
	hM3Dq+NS, n=7			p=0.6824	0.9448	\
	hM3Dq+CNO, n=9			p=0.2607	0.9015	\
	mCherry+NS, n=7;<br>mCherry+CNO, n=7;<br>hM3Dq+NS, n=7;<br>hM3Dq+CNO, n=9.		Two-way ANOVA,<br>Tukey’s multiple comparison test.	\	\	Interaction:F(1,26)=2.915,P=0.0997<br>Treatment:F(1,26)=4.687,P=0.0398<br>Group:F(1,26)=9.354,P=0.0051<br>multiple comparisons:<br>NS(mCherry vs. hM3Dq), p=0.9890;<br>CNO(mCherry vs. hM3Dq), p=0.0421*;<br>mCherry(NS vs. CNO), p=0.7900;<br>hM3Dq(NS vs. CNO), p=0.0092**.
Figure 4E	mCherry+NS, n=7		Normality test_Shapiro-Wilk (W)	p=0.2736	0.8898	\
	mCherry+CNO, n=7			p=0.6168	0.9376	\
	hM3Dq+NS, n=7			p=0.4505	0.9175	\
	hM3Dq+CNO, n=9			p=0.2369	0.8973	\
	mCherry+NS, n=7;<br>mCherry+CNO, n=7;<br>hM3Dq+NS, n=7;<br>hM3Dq+CNO, n=9.		Two-way ANOVA,<br>Tukey’s multiple comparison test.	\	\	Interaction:F(1,26)=5.355,P=0.0288<br>Treatment:F(1,26)=4.036,P=0.0550<br>Group:F(1,26)=3.496,P=0.0728<br>multiple comparisons:<br>NS(mCherry vs. hM3Dq), p=0.9967;<br>CNO(mCherry vs. hM3Dq), p=0.0199*;<br>mCherry(NS vs. CNO), p=0.9899;<br>hM3Dq(NS vs. CNO), p=0.0252*.
	mCherry+NS, n=7			p=0.0347	0.7928	\

Figure 4F	mCherry+CNO, n=7<br>hM3Dq+NS, n=7<br>hM3Dq+CNO, n=9	Normality test_Shapiro-Wilk (W)	p=0.2311<br>p=0.0195<br>p=0.7573	0.881<br>0.7679<br>0.9562	\<br>\<br>\
	mCherry+NS, n=7<br>mCherry+CNO, n=7<br>hM3Dq+NS, n=7<br>hM3Dq+CNO, n=9	Nonparametric test	NS(mCherry vs.hM3Dq), p=1.000<br>CNO(mCherry vs.hM3Dq), p=0.101<br>mCherry(NS vs.CNO), p=1.000<br>hM3Dq(NS vs.CNO), p=0.020*	\<br>\<br>\<br>\	\<br>\<br>\<br>\
Figure 4G	mCherry+NS, n=7<br>mCherry+CNO, n=7<br>hM3Dq+NS, n=7<br>hM3Dq+CNO, n=9	Normality test_Shapiro-Wilk (W)	p=0.0616<br>p=0.1444<br>p=0.2939<br>p=0.0077	0.8181<br>0.8577<br>0.8936<br>0.763	\<br>\<br>\<br>\
	mCherry+NS, n=7<br>mCherry+CNO, n=7<br>hM3Dq+NS, n=7<br>hM3Dq+CNO, n=9	Nonparametric test	NS(mCherry vs.hM3Dq), p=1.000<br>CNO(mCherry vs.hM3Dq), p=0.050*<br>mCherry(NS vs.CNO), p=1.000<br>hM3Dq(NS vs.CNO), p=0.098	\<br>\<br>\<br>\	\<br>\<br>\<br>\
Figure 4I	mCherry+NS, n=7<br>mCherry+CNO, n=7<br>hM3Dq+NS, n=7<br>hM3Dq+CNO, n=9	Normality test_Shapiro-Wilk (W)	p=0.3863<br>p=0.6066<br>p=0.4852<br>p=0.0975	0.9086<br>0.9364<br>0.922<br>0.8607	\<br>\<br>\<br>\
	mCherry+NS, n=7;<br>mCherry+CNO, n=7;<br>hM3Dq+NS, n=7;<br>hM3Dq+CNO, n=9.	Two-way ANOVA,<br>Tukey's multiple comparison test.	\	\	Interaction:F(1,26)=0.03644,P=0.8501<br>Treatment:F(1,26)=0.7741,P=0.3870<br>Group:F(1,26)=0.1458,P=0.7057<br>multiple comparisons:<br>NS(mCherry vs. hM3Dq), p=0.8818;<br>CNO(mCherry vs. hM3Dq), p=0.9578;<br>mCherry(NS vs. CNO), p=0.9789;<br>hM3Dq(NS vs. CNO), p=0.9990.
Figure 4J	mCherry+NS, n=7<br>mCherry+CNO, n=7<br>hM3Dq+NS, n=7<br>hM3Dq+CNO, n=9	Normality test_Shapiro-Wilk (W)	p=0.3072<br>p=0.1781<br>p=0.5295<br>p=0.417	0.896<br>0.8679<br>0.9275<br>0.9229	\<br>\<br>\<br>\
	mCherry+NS, n=7;<br>mCherry+CNO, n=7;<br>hM3Dq+NS, n=7;<br>hM3Dq+CNO, n=9.	Two-way ANOVA,<br>Tukey's multiple comparison test.	\	\	Interaction:F(1,26)=0.03644,P=0.8501<br>Treatment:F(1,26)=0.7741,P=0.3870<br>Group:F(1,26)=0.1458,P=0.7057<br>multiple comparisons:<br>NS(mCherry vs. hM3Dq), p=0.9996;<br>CNO(mCherry vs. hM3Dq), p=0.0276*;<br>mCherry(NS vs. CNO), p=0.9996;<br>hM3Dq(NS vs. CNO), p=0.0456*.
	mCherry+NS, n=7<br>mCherry+CNO, n=7<br>hM4Di+NS, n=7<br>hM4Di+CNO, n=9	Normality test_Shapiro-Wilk (W)	p=0.1968<br>p=0.5493<br>p=0.7336<br>p=0.0031	0.8729<br>0.9298<br>0.9504<br>0.7291	\<br>\<br>\<br>\

Figure 5D	mCherry+NS, n=7;<br>mCherry+CNO, n=7;<br>hM4Di+NS, n=7;<br>hM4Di+CNO, n=9.	Two-way ANOVA,<br>Tukey's multiple comparison test.	\	\	Interaction:F(1,26)=0.7382,P=0.3981<br>Treatment:F(1,26)=0.09013,P=0.7664<br>Group:F(1,26)=1.361,P=0.2539<br>multiple comparisons:<br>NS(mCherry vs. hM4Di), p=0.8553;<br>CNO(mCherry vs. hM4Di), p=0.9767;<br>mCherry(NS vs. CNO), p=0.5153;<br>hM4Di(NS vs. CNO), p=0.9959.
Figure 5E	mCherry+NS, n=7<br>mCherry+CNO, n=7<br>hM4Di+NS, n=7<br>hM4Di+CNO, n=9	Normality test_Shapiro-Wilk (W)	p=0.3833<br>p=0.743<br>p=0.7257<br>p=0.8683	0.9082<br>0.9515<br>0.9496<br>0.967	\<br>\<br>\<br>\
	mCherry+NS, n=7;<br>mCherry+CNO, n=7;<br>hM4Di+NS, n=7;<br>hM4Di+CNO, n=9.	Two-way ANOVA,<br>Tukey's multiple comparison test.	\	\	Interaction:F(1,26)=16.04,P=0.0005<br>Treatment:F(1,26)=9.707,P=0.0044<br>Group:F(1,26)=6.805,P=0.0149<br>multiple comparisons:<br>NS(mCherry vs. hM4Di), p=0.9277;<br>CNO(mCherry vs. hM4Di), p=0.0001***;<br>mCherry(NS vs. CNO), p=0.7732;<br>hM4Di(NS vs. CNO), p=0.0003***.
Figure 5F	mCherry+NS, n=7<br>mCherry+CNO, n=7<br>hM4Di+NS, n=7<br>hM4Di+CNO, n=9	Normality test_Shapiro-Wilk (W)	p=0.1228<br>p=0.3001<br>p=0.7625<br>p=0.0546	0.8499<br>0.8947<br>0.9536<br>0.8378	\<br>\<br>\<br>\
	mCherry+NS, n=7;<br>mCherry+CNO, n=7;<br>hM4Di+NS, n=7;<br>hM4Di+CNO, n=9.	Two-way ANOVA,<br>Tukey's multiple comparison test.	\	\	Interaction:F(1,26)=11.88,P=0.0019<br>Treatment:F(1,26)=4.691,P=0.0019<br>Group:F(1,26)=3.174,P=0.0865<br>multiple comparisons:<br>NS(mCherry vs. hM4Di), p=0.8150;<br>CNO(mCherry vs. hM4Di), p=0.0020**;<br>mCherry(NS vs. CNO), p=0.6660;<br>hM4Di(NS vs. CNO), p=0.0040**.
Figure 5G	mCherry+NS, n=7<br>mCherry+CNO, n=7<br>hM4Di+NS, n=7<br>hM4Di+CNO, n=9	Normality test_Shapiro-Wilk (W)	p=0.0861<br>p=0.0082<br>p=0.0009<br>p=0.0077	0.8334<br>0.732<br>0.6462<br>0.763	\<br>\<br>\<br>\
	mCherry+NS, n=7<br>mCherry+CNO, n=7<br>hM4Di+NS, n=7<br>hM4Di+CNO, n=9	Nonparametric test	NS(mCherry vs.hM4Di), p=1.000<br>CNO(mCherry vs.hM4Di), p=0.021*<br>mCherry(NS vs.CNO), p=1.000<br>hM4Di(NS vs.CNO), p=0.003**	\<br>\<br>\<br>\	\<br>\<br>\<br>\
	mCherry+NS, n=7<br>mCherry+CNO, n=7<br>hM4Di+NS, n=7<br>hM4Di+CNO, n=9	Normality test_Shapiro-Wilk (W)	p=0.8994<br>p=0.0879<br>p=0.6479<br>p=0.8054	0.9701<br>0.8343<br>0.941<br>0.9607	\<br>\<br>\<br>\

Figure 5I	mCherry+NS, n=7;<br>mCherry+CNO, n=7;<br>hM4Di+NS, n=7;<br>hM4Di+CNO, n=9.	Two-way ANOVA,<br>Tukey's multiple comparison test.	\	\	Interaction:F(1,26)=2.490,P=0.1266<br>Treatment:F(1,26)=0.2186,P=0.6440<br>Group:F(1,26)=1.366,P=0.2530<br>multiple comparisons:<br>NS(mCherry vs. hM4Di), p=0.5072;<br>CNO(mCherry vs. hM4Di), p=0.8494;<br>mCherry(NS vs. CNO), p=0.9921;<br>hM4Di(NS vs. CNO), p=0.2131.
Figure 5J	mCherry+NS, n=7<br>mCherry+CNO, n=7<br>hM4Di+NS, n=7<br>hM4Di+CNO, n=9	Normality test_Shapiro-Wilk (W)	p=0.3863<br>p=0.6066<br>p=0.4852<br>p=0.0975	0.9086<br>0.9364<br>0.922<br>0.8607	\<br>\<br>\<br>\
	mCherry+NS, n=7;<br>mCherry+CNO, n=7;<br>hM4Di+NS, n=7;<br>hM4Di+CNO, n=9.	Two-way ANOVA,<br>Tukey's multiple comparison test.	\	\	Interaction:F(1,26)=5.895,P=0.0224<br>Treatment:F(1,26)=1.878,P=0.1823<br>Group:F(1,26)=6.702,P=0.0156<br>multiple comparisons:<br>NS(mCherry vs. hM4Di), p=0.8855;<br>CNO(mCherry vs. hM4Di), p=0.0473*;<br>mCherry(NS vs. CNO), p=0.9995;<br>hM4Di(NS vs. CNO), p=0.0059**.
