# Supplementary material for: Amygdalo‐nigral circuit mediates stress‐induced vulnerability to the parkinsonian toxin MPTP
Source: CNS Neurosci Ther. 2023 Mar 13;29(7):1940–52. doi: 10.1111/cns.14151 (PMC10324352; doi:10.1111/cns.14151)
Supplement: Supplementary file 2 — Appendix S1. [file CNS-29-1940-s002.docx]

**Amygdalo-nigral circuit mediates stress-induced vulnerability to the parkinsonian toxin MPTP**

Hongwei Cai^1, 2^, Pei Zhang^1, 3, 4^, Tongxia Li^1^, Ming Li^1^, Lijun Zhang^1^, Chi Cui^1^, Jie Lei^1^, Jian Yang^1^, Kun Ren^1^, Jie Ming^5^ ^*^, Bo Tian^1, 3, 4 *^

*^1^ Department of Neurobiology, School of Basic Medicine, Tongji Medical College, Huazhong University of Science and Technology, Wuhan, Hubei, P. R. China*

*^2^ Clinical College of Traditional Chinese Medicine, Hubei University of Chinese Medicine, Wuhan, Hubei, P. R. China*

*^3^ Institute for Brain Research, Huazhong University of Science and Technology, Wuhan, Hubei, P. R. China*

*^4^ Key Laboratory of Neurological Diseases, Ministry of Education, Wuhan, Hubei, P. R. China*

*^5^ Department of Breast and Thyroid Surgery, Union Hospital, Tongji Medical College, Huazhong University of Science and Technology, Wuhan, Hubei, P. R. China*

**^*^ Correspondence to:** Dr. Jie Ming, Department of Breast and Thyroid Surgery, Union Hospital, Tongji Medical College, Huazhong University of Science and Technology, No. 1277 Jiefang Avenue, Wuhan, Hubei 430030, P. R. China. E-mail: [mingjiewh@126.com](mailto:mingjiewh@126.com) and Dr. Bo Tian, Department of Neurobiology, School of Basic Medicine, Tongji Medical College, Huazhong University of Science and Technology, No.13 Hangkong Road, Wuhan, Hubei 430030, P. R. China; E-mail: [tianbo@mails.tjmu.edu.cn](mailto:tianbo@mails.tjmu.edu.cn).

Hongwei Cai and Pei Zhang contributed equally.

**Supplementary methods**

**Social interaction test**

For the first phrase, Mice were introduced into an open field area (50 cm × 50 cm × 50 cm) with an empty transparent perforated cage (6.5 cm width × 10 cm length × 42 cm height) for 150s (No target). For the second phrase，repeating the first phrase but with an aggressive CD1 in the transparent perforated cage (Target). The time of mice spent in the interaction zone surrounded the perforated cage was record. The social interaction ratio (SIR) was calculated by time in the interaction zone with target / time in the interaction zone with no target. SIR > 1 was defined as resistant mice，while SIR > 1 as susceptible mice. Only susceptible mice entered following experiments.

**Open field test**

An open field area was made of white PVC (50cm × 50cm × 50cm), and the floor of the box was divided into 25 grids of 10cm × 10cm. First, mice were allowed to freely explore for 5min to habituate. Then recording animals’ spontaneous activity for 10 min. A video-tracking system (SuperMaze, Xinruan, Shanghai, China) was used to record and analysis time spent in the center area of the open field.

**Elevated plus maze test**

The EPM consisted of two open arms, two closed arms with gray walls, and a central platform. Each arm is 350mm length with 100mm aboveground. Mice were allowed 2min to habituate in the EPM. When the test started, mice were first placed in the central platform, then recording animals’ activity in EPM for 5min by the video-tracking system (SuperMaze, Xinruan, Shanghai, China). The time in open arms was analyzed to value animals’ performance.

**Tail suspension test**

Mice were suspended using a piece of adhesive tape for 5min. The immobility time that considered as an index of depressive-like behavior was measured. The chambers were cleaned with 75% alcohol between each test.

**Forced swimming test**

In the forced swimming test, mice were first placed in a cylinder with enough water so that they couldn’t touch the bottom with its hind paws. Then “struggle”, “swim”, and “immobile” postures were recorded by the video-tracking system for 5min. The time of immobility was calculated to assess animals’ depressive state.

**Locomotion test**

The locomotion test was operated in the open field device. The day before the test day, mice were pre-adapted to the field for 5min. When the test began, mice were placed in the center of the field and recorded for 10min through the camera-tracking system. The open field area was cleaned after each trail. The distance traveled in 10min was finally analyzed.

**Rotarod test**

The rotarod test was used to evaluated animals’ motor coordination as described previously^23^. Before test, all mice were trained on the rotarod (diameter = 6cm, 5 to 10 rpm/min in 300s) for 3 rounds. On the test day, the rotarod test was conducted at an accelerating speed from 5 to 40 rpm/min in 300s for three rounds. The device was cleaned with 75% alcohol between each trail. finally, the average latency to fall was recorded and analyzed.

**Pole test**

In the pole test, the apparatus consisted of a wooden pole and a ball on the top of the pole. The pole was 70cm high, 0.5cm diameter, and was wrapped with gauze to prevent mice slipping. At the bottom of the pole was a bedding to protect mice from injury. During the pole test, the total time it took for mice to get from the top to the bottom was measured.

**Ledge test**

Mouse sensory motor capability was usually assessed by the ledge test. This measure is the most directly comparable to human signs of ataxia. First, lift mice from their cages and put it on the cages’ ledge. Then observe mice walking along the cage ledge and score their performance. A score of 0 was assigned to the mice that kept balance on the ledge and lowered themselves back into the cage gracefully. A score of 1 was assigned to the mice that lost their foot while walking along the ledge without other omission. If mice didn't use their hind paws effectively and landed on the bottom of the cage with head rather than paws, they received a score of 2. If mice fell from the ledge or refused to move on with shake, they were scored 3.

**Viral preparations**

Recombinant AAV vectors and rabies were used for delivering target genes: AAV9-TH-Cre, AAV9-DIO-G, AAV9-DIO-TVA-EYFP, RV-EnVA-ΔG-dsRed, RV-EnVA-ΔG-Gcamp6s-dsRed, AAV1-TH-Cre, AAV9-DIO-hM3Dq-mCherry, AAV9-DIO-hM4Di-mCherry. Viral vectors were subdivided into aliquot stored at −80 °C until use. All the virus vectors were purchased from BrainVTA (BrainVTA Co., Ltd., Wuhan, China).

**Stereotaxic surgery**

Mice were first deeply anesthetized by sodium pentobarbital (70 mg/kg, intraperitoneal injection). Then mice were removed hair on the head and fixed on the stereotaxic instrument (RWD, China). The head was sterilized with iodophor and skull was exposed by operating scissors. The target brain regions were located by stereotaxic apparatus (RWD, China). Injections were next performed via a 10ml micro-syringe (WPI, USA) and a micro-syringe pump (Stoelting, USA). Viruses were delivered at a rate of 30 nl/min. After injection, the needle was retained in the target area for 10min to prevent virus reflux. Finishing the craniotomy, the incision was closed and animals were put on the heating cushions until awake. The injected coordinates were SNc (from lambda: AP ±1.5mm, ML +1.2mm, DV -4.3mm), and CeA (from bregma: AP ±2.85mm, ML -1.4mm, DV -4.73mm).

**Immunofluorescence staining**

Mouse brains were fixed in 4% paraformaldehyde, and then coronally sectioned in 30μm via cryostat microtome. These sections were washed with PBS three times. 1% sodium dodecyl sulphate were used to antigen retrieval and 0.5% Triton-X were used to rupture membrane. Non-specific binding sites were blocked with 10% goat serum for 1h. Then sections were incubated with rabbit polyclonal anti-TH (1:200, 25859-1-AP, Proteintech) overnight at 4℃. After rinsing three times in PBS, sections were incubated for 1h in fluorochrome-conjugated secondary antibody (1:400, Dylight-488-labelled goat anti-rabbit, Abbikine, CA, USA) away from light. Washed in the PBS for three times, sections were stored at 4℃ before analysis. Expression of TH were imaged by Olympus IX-73 microscope (Olympus, Japan).

**Statistical analysis**

All statistics were performed with GraphPad Prism 7 software (GraphPad Software, Inc., San Diego, CA) or MATLAB. The Shapiro-Wilk normality test was conducted before choosing the appropriate statistical test. The Group differences were determined by either two-tailed Student’s t-test, ordinary one-way ANOVA with Dunnett's multiple comparisons test, or two-way ANOVA with post hoc Tukey’s multiple comparisons test depending on the data type property. Data were expressed as mean ± SEM and the point of significance was set as ^*^P ≤ 0.05, ^**^P ≤ 0.01, ^***^P ≤ 0.001, ^****^P ≤ 0.0001. All detailed statistics are showed in the file of Statistical Report.
